# Preventing and Treating Insomnia Symptoms in Midlife and Older Adults (ASLEEP): Protocol for a Randomized Controlled Trial Using the PROTECT Norge Infrastructure

**DOI:** 10.2196/81542

**Published:** 2026-03-02

**Authors:** Jon Arild Aakre, Bjørn Bjorvatn, Martha Therese Gjestsen, Ingvild Dalen, Clive Ballard, Ingelin Testad

**Affiliations:** 1 Centre for Age-Related Medicine - SESAM, Stavanger University Hospital, Jan Johnsens gate nr 16, Stavanger, 4011, Norway, 47 48281559; 2 Department of Clinical Medicine, University of Bergen, Bergen, Norway; 3 Department of Global Public Health and Primary Care, University of Bergen, Bergen, Norway; 4 Norwegian Competence Center for Sleep Disorders, Haukeland University Hospital, Bergen, Norway; 5 Department of Research, Stavanger University Hospital, Stavanger, Norway; 6 Department of Clinical Biosciences, Faculty of Health and Life Sciences, University of Exeter, Exeter, United Kingdom; 7 Department of Health and Community Sciences, Faculty of Health and Life Sciences, University of Exeter, Exeter, United Kingdom

**Keywords:** insomnia symptoms, cognitive behavioral therapy for insomnia, eHealth, randomized controlled trial, older adults, sleep intervention, PROTECT Norge

## Abstract

**Background:**

Sleep is increasingly recognized as a fundamental determinant of health and brain function. Sleep difficulties are common in older adults, with a substantial proportion reporting problems initiating or maintaining sleep, which can negatively affect mental and physical health, cognitive function, and quality of life. Cognitive behavioral therapy for insomnia (CBT-I) is the gold-standard treatment for insomnia disorder; however, its reach is limited due to resource demands and a shortage of professionals that can deliver it. Digitally delivered CBT-I via eHealth platforms increases accessibility and has demonstrable effects but remains limited in many countries.

**Objective:**

The objective of this paper is to describe the protocol for the further development and evaluation of ASLEEP (Preventing and Treating Insomnia Symptoms in Midlife and Older Adults), a tiered, digitally delivered CBT-I intervention designed to reduce insomnia severity and improve related health outcomes in adults aged 50 years and older.

**Methods:**

The project will be conducted in 2 phases. Phase 1 focuses on refining and optimizing ASLEEP, developing an advanced CBT-I course, and integrating a nested trial into PROTECT (Platform for Research Online to investigate Cognition and Genetics in Ageing) Norge, a fully automated digital research platform. Phase 2 is a fully digital, 2-arm, waitlist-controlled randomized controlled trial, with 400 participants randomized 1:1 to the intervention or waitlist control and allocation stratified by age and insomnia severity. Outcomes will be assessed at baseline and at 3, 6, and 12 months, with a 15-month follow-up for the waitlist group. The primary outcome is insomnia severity measured by the Insomnia Severity Index. Secondary outcomes include sleep medication use, depression, anxiety, and cognition.

**Results:**

The project started in January 2026, with funding awarded. As of February 2026, phase 1—intervention optimization and development—is underway. Ethics approval for ASLEEP has not been submitted. Following completion of phase 1, phase 2, which includes a digital randomized controlled trial, will commence; as of February 2026, no participants have been recruited, and data collection and data analysis have not yet started. Short-term data collection is planned to be completed by summer 2028, with results disseminated in winter 2028.

**Conclusions:**

This trial will evaluate the short- and long-term effectiveness of a tiered digital CBT-I intervention for midlife and older adults. By leveraging the PROTECT Norge platform and if effective, ASLEEP may represent a scalable model for low-threshold, accessible prevention and treatment of symptoms of insomnia.

## Introduction

### The Role of Sleep in Healthy Aging and Brain Health

Sufficient nighttime sleep is vital for maintaining optimal daytime functioning and supporting overall health and quality of life. For most adults, this generally involves obtaining 6 to 9 hours of sleep per night [1]. With increasing age, meeting individual sleep needs in terms of both duration and quality becomes harder [2], and up to 50% of older adults report difficulties initiating or maintaining sleep [3]. Failure to meet sleep needs and chronic sleep deprivation impair mood and emotional regulation; disrupt multiple physiological systems and cognitive functions; weaken immune responses; and increase the risk of several noncommunicable diseases, including cardiovascular disease, diabetes, and obesity, as well as mental health disorders [4]. Beyond these individual consequences, sleep deprivation also carries societal costs, contributing to increased health care expenditures, reduced productivity, and higher rates of workforce absenteeism, collectively amounting to 1% to 3% of gross domestic product in OECD countries [5].

Poor sleep is associated with poorer brain health in midlife, suggesting that sleep is a potential target for maintaining brain health into older age [6]. Experimental studies have shown that sleep deprivation impairs the efficiency of the glymphatic system, a key pathway for clearing neurotoxic waste products from the brain during sleep [7]. This impairment has been associated with β-amyloid deposition in older adults, which may in turn contribute to Alzheimer disease risk [8]. These mechanistic insights provide one of several pathways that may help explain the observed epidemiological association between sleep deprivation in midlife and an increased incidence of dementia later in life [9]. Beyond experimental and epidemiological evidence, sleep is increasingly recognized as a vital public health priority [4] and has been formally positioned as an equal pillar of health alongside physical activity and nutrition [10], underscoring its essential role in maintaining both brain function and overall health and well-being.

### When Poor Sleep Develops Into Insomnia: Insomnia Treatment Using Cognitive Behavioral Therapy

Difficulties falling asleep or staying asleep, or waking too early are the defining symptoms of insomnia. Moreover, a symptom burden with a frequency equal to or exceeding 3 days per week, persisting for over 3 months, resulting in daytime impairment, warrants a diagnosis of chronic insomnia [11]. While general sleep deprivation is associated with widespread physiological and cognitive consequences across multiple systems, insomnia is notably characterized by a strong and well-documented bidirectional relationship with mental health conditions, particularly anxiety and depression [12]. According to the Harvey cognitive model, insomnia is not just a consequence of insufficient sleep; rather, maladaptive thoughts, heightened arousal, and counterproductive behaviors interact to maintain the disorder through a feedback loop of worry, arousal, and behaviors that make sleep more difficult [13]. Building on this model, cognitive behavioral therapy for insomnia (CBT-I) is currently the gold-standard treatment for chronic insomnia and is a highly effective and safe treatment [11]. CBT-I combines behavioral strategies (eg, sleep restriction, stimulus control, and relaxation), cognitive techniques (eg, restructuring unhelpful thoughts, mindfulness, and paradoxical intention), and educational elements, including psychoeducation and sleep hygiene, and is usually delivered by trained professionals, such as psychologists or psychiatrists, over several structured sessions tailored to the patient’s needs [14]. It is a resource-demanding treatment, and combined with a lack of professionals with the competency to deliver CBT-I, it is usually reserved for those with a severe diagnosis of insomnia [15]. In Norway, Professor Bjørn Bjorvatn has published a CBT-I–based self-help book targeting adults with insomnia. In a randomized controlled trial (RCT) among self-referred participants, the tools and strategies presented in the book improved sleep, reduced use of sleep medication, and slightly decreased depressive symptoms compared with standard sleep hygiene advice [16]. A subsequent RCT among general practitioner–referred patients showed similar benefits, including reductions in sleep medication use and anxiety, as well as improved sleep outcomes [17]. These studies indicate that the self-help book is an effective low-threshold intervention for adults with insomnia in both community and primary care settings.

### eHealth Platforms for Increasing the Reach and Dissemination of CBT-I

The continuing digitization of people’s daily life has paved the way for new platforms delivering CBT-I via the internet (eCBT-I). eCBT-I offers wide reach and accessibility and serves as an effective alternative or supplement to traditional, in-person-delivered CBT-I [18]. However, access to evidence-based, eCBT-I programs, such as SLEEPIO [19] and Somryst [20], which are available by prescription in some health care systems, remains limited in many countries, including Norway.

The ASLEEP project (Preventing and Treating Insomnia Symptoms in Midlife and Older Adults) aims to enhance access to sleep education and insomnia symptom treatment by leveraging eHealth technologies. The intervention is based on the aforementioned CBT-I–based self-help book [16] and is tailored to the needs of an aging population through a tiered delivery model. The project is supported by the Platform for Research Online to Investigate Cognition and Genetics in Ageing Norge (PROTECT Norge) [21], which was initiated by the Centre for Age-Related Medicine (SESAM) at Stavanger University Hospital in 2020. PROTECT Norge is a digital, prospective research cohort with yearly follow-ups. Focusing on the brain aging, it assesses physical and mental health and lifestyle factors through validated questionnaires, as well as cognitive function using a neuropsychiatric test battery. In addition, the PROTECT infrastructure, originating from University of Exeter and Kings College London in the UK, includes a built-in research infrastructure designed to support large-scale digital RCTs [22,23]. The PROTECT Norge cohort [21] currently consists of 5547 registered individuals, who primarily have been recruited through social media, particularly Facebook. Interested individuals self-refer and are directed to the PROTECT Norge study website [24], where they complete eligibility screening, registration, and informed consent through a secure, fully automated online process. Once enrolled, participants complete annual validated cognitive and mental health assessments for up to 10 years and provide detailed information on lifestyle, medical, and demographic factors. Participants are also asked to provide consent for contact (C4C), which has been granted by 94% of PROTECT Norge participants [21]. Combining this with the PROTECT research infrastructure, which enables the conduction of nested studies, PROTECT Norge is well placed to deliver large-scale interventions and clinical trials in a time- and resource-efficient manner within the PROTECT Norge study trajectory.

To prevent the development of insomnia and promote good sleep, the ASLEEP intervention offers a basic course (tier 1). Supported by the PROTECT Norge infrastructure, a screening procedure will be developed to flag individuals with symptoms of insomnia of a degree that suggest the need for more intensive eCBT-I treatment and to guide those individuals to an advanced course (tier 2). The PROTECT Norge research infrastructure has already had its feasibility of delivering ASLEEP as a nested study in PROTECT Norge assessed, and the acceptability of the basic course content was also evaluated in the same study by this group (JA Aakre, PhD; I Testad, PhD; MT Gjestsen, PhD; et al; unpublished data; November 2025).

This paper describes the protocol for the ASLEEP project, including further development of the ASLEEP intervention to include an advanced course and its digital delivery (phase 1), as well as the evaluation of its effectiveness (phase 2) through a 2-arm RCT, with a waitlist design. The RCT will be conducted entirely digitally and facilitated by the PROTECT Norge research infrastructure.

The objectives of the ASLEEP project are to:

1. Further develop the ASLEEP intervention, an eCBT-I–based, tiered sleep program, including an advanced course aimed at reducing insomnia symptoms in adults aged 50 years and older.
2. Conduct short- and long-term evaluations using the PROTECT Norge infrastructure to assess the effectiveness of the ASLEEP advanced course on sleep-related outcomes among midlife and older adults.
3. Evaluate the effectiveness of ASLEEP on established outcomes that exist concomitantly or are related to insomnia symptoms, such as medication use, depression, anxiety, and cognitive function.

### Hypotheses

Our first hypothesis (H1) is that further development of the ASLEEP intervention—an eCBT-I–based, tiered sleep program—and its subsequent dissemination within the PROTECT Norge cohort will lead to a significant reduction in insomnia symptoms among individuals aged 50 years and older compared with waitlist control group.

Our second hypothesis (H2) is that ASLEEP will result in short-term improvements in sleep outcomes (Insomnia Severity Index [ISI] score and sleep medication use) among midlife and older adults, and these improvements will be sustained in the long-term.

Our third hypothesis (H3) is that ASLEEP will not only improve sleep outcomes but also lead to significant reductions in symptoms of depression, anxiety, and cognitive decline among midlife and older adults compared to a waitlist control group.

## Methods

### Reporting Guidelines

This protocol is reported in accordance with the Guidance for Reporting Involvement of Patients and the Public–Short Form (GRIPP2-SF) checklist [25] and the SPIRIT 2025 (Standard Protocol Items: Recommendations for Interventional Trials) guidelines (See Checklist 1 and Checklist 2).

### Intervention

Following development conducted in phase 1 of the project, the ASLEEP intervention will consist of 2 courses. The basic course has been developed to promote good sleep in a broad group of adults aged 50 years and older (JA Aakre, PhD; I Testad, PhD; MT Gjestsen, PhD; et al; unpublished data; November 2025) and starts off with a video providing instructions on how to complete the digital sleep diary. Participants will then complete a 7-day sleep diary. After completion of the sleep diary, 3 educational modules will be unlocked and delivered through short videos (see Table 1 for video descriptions) focusing on healthy sleep and sleep habits. Then, feedback reports based on recorded sleep through the sleep diary will be made available to participants, with a focus on sleep efficiency. Findings from the acceptability and feasibility study (JA Aakre, PhD; I Testad, PhD; MT Gjestsen, PhD; et al; unpublished data; November 2025) showed that the basic course content was well accepted, with participants describing the videos as clear, systematic, appropriately lengthy, and helpful for understanding sleep and their own sleep patterns. Several participants appreciated the expert-led dialogue format of the videos. The sleep diary was also viewed as meaningful, increasing awareness of sleep habits and prompting daily reflection on factors affecting sleep.

**Table 1.** Video descriptions.

Video (length)	Name	Description
Sleep diary instruction (17 min 17 s)	An instruction to keeping digital sleep diary	This video provides step-by-step instructions on how to keep a sleep diary, along with clarification of common misunderstandings people may have when logging their sleep.
Video module 1 (20 min 3 s)	What is sleep and how it is regulated	This video explains what sleep is, its role in the body, and the biological processes that govern it.
Video module 2 (18 min 10 s)	Assessing poor sleep	This video addresses how poor sleep can be assessed, discusses its possible causes, approaches to diagnosis, and provides a general overview of different sleep disorders.
Video module 3 (30 min 10 s)	Treatment of insomnia	This video module focuses on the management of insomnia, including definitions, prevalence, and treatment strategies for both short-term and chronic insomnia, as well as principles of sleep regulation. Additionally, Professor Bjorvatn gives sleep hygiene advice, discussing ways to maintain sufficient sleep, support a healthy circadian rhythm, and minimize evening and nighttime arousal through habits and behavioral practices.

As part of phase 1 of the project, the ASLEEP advanced course will be developed and subsequently offered to participants in a fully digital RCT to assess its effect on sleep outcomes. Eligible participants who have completed the basic course will be funneled into the advanced course if their post–basic course score on the ISI [25] is 10 or higher (defined as symptoms of insomnia) [26].

The advanced course will be developed according to established protocol for CBT-I, drawing on core components such as sleep restriction, stimulus control, cognitive restructuring, sleep-hygiene reinforcement, and relapse prevention [27]. However, unlike other existing digital CBT-I programs (eg, SHUTi, Sleepio), which deliver these components within a single treatment stream, ASLEEP applies a stepped-care approach [28] in which only participants who continue to report insomnia symptoms after completing the basic course progress to the more treatment-intensive modules. The ASLEEP advanced course is expected to consist of a series of weekly digital modules delivered over approximately 6 weeks, although the exact number and pacing of modules will be finalized during the development phase. Consistent with evidence identifying sleep restriction and stimulus control as the most effective components of CBT-I [29], these components will constitute the core of the intervention. Sleep restriction aims to consolidate sleep and strengthen homeostatic sleep pressure by limiting time in bed to approximate actual sleep duration and gradually expanding it as sleep becomes more efficient [28]. Stimulus control complements this by re-establishing the bed and bedroom as cues for sleep rather than wakefulness through structured behavioral instructions, including going to bed only when sleepy, leaving the bed if unable to sleep, and maintaining consistent rise times.

A sleep diary will be completed throughout the advanced course to track progress and to tailor individual intervention recommendations, including ongoing adjustment of the sleep-restriction schedule.

### Phase 1: ASLEEP Development, Implementation, and Integration

The ASLEEP project is guided by the updated Medical Research Council (MRC) framework for the development and evaluation of complex interventions, which emphasizes an iterative process integrating development, feasibility testing, evaluation, and implementation, supported by ongoing consideration of context, program theory, stakeholder engagement, and economic factors [30]. In line with this framework, we have already developed (phase 1 of the MRC framework) and assessed the acceptability of the basic course and the feasibility of delivering ASLEEP through the PROTECT Norge infrastructure (phase 2 of the MRC framework) (JA Aakre, PhD; I Testad, PhD; MT Gjestsen, PhD; et al; unpublished data; November 2025), and we are moving on to the next phases of development and evaluation.

Building on the findings from the acceptability and feasibility study showing that some participants missed the opportunity to use their own devices rather than a study-provided iPad (JA Aakre, PhD; I Testad, PhD; MT Gjestsen, PhD; et al; unpublished data; November 2025), the user interface will be optimized to support access across devices, including smartphones, tablets, and personal computers, via both a mobile app or a web browser. This process will be informed by human-centered design to ensure user and stakeholder engagement in the development process. While the MRC framework provides overarching guidance for complex intervention development and evaluation, design thinking adds a complementary methodological perspective to the development phase of the project.

Integration of the ASLEEP study, including registration, consenting and screening, web page text, study documents, and new questionnaires on the PROTECT Norge infrastructure, will undergo multiple rounds of user acceptance testing (UAT) in a process described in a previous publication.

As part of the implementation work, a dialogue with the Western Regional Health Authority digitalization department has been initiated to discuss technical compatibility and potentially building on existing regional digital health infrastructures, thereby supporting potential future integration into existing health care pathways and real-world deployment.

### Phase 2: Short- and Long-Term Evaluation of ASLEEP

#### RCT Design

This study will be an RCT nested within the cohort of PROTECT Norge, a 10-year digital follow-up study with annual data collection since the start in 2020. The treatment arm will be compared with a waitlist control group. Screening, recruitment, and data collection will be conducted through the PROTECT platform. This RCT will be registered in ClinicalTrials.gov.

### Comparison Group

Eligible participants that are not randomized to the intervention will go into a waitlist control group and will receive access to the intervention after a predetermined delay.

### Recruitment

Participants will be recruited from the PROTECT Norge cohort among individuals who have provided consent for contact regarding future studies. A general invitation will be sent to the whole cohort to perform the basic (tier 1) ASLEEP course. Subsequent scoring of the ISI [26] score and the Global Sleep Assessment Questionnaire [31] will be used to evaluate eligibility for the advanced (tier 2) ASLEEP course and hence the RCT (see further details below).

### Eligibility Criteria

Inclusion criteria are as follows: participants must have completed the ASLEEP basic course, have an ISI score of 10 or higher, and have an insomnia duration exceeding 3 months.

Exclusion criteria are as follows: participants with insomnia symptoms that stem from other sleep disorders, including obstructive sleep apnea, restless legs syndrome, parasomnias, and hypersomnia, will be screened for based on selected items (Q2, Q6, Q7, and Q9) from the Global Sleep Assessment Questionnaire [32].

### Randomization and Masking

Participants will be randomized in a 1:1 ratio to either the intervention group or the waitlist control group using simple block randomization with varying block sizes of 6 to 10 participants. The allocation sequence will be computer generated in advance by a member of the research team not involved in participant recruitment or follow-up.

Randomization will be performed after completion of baseline assessments and confirmation of eligibility. Allocation will be implemented manually by authorized PROTECT Norge study staff using the pregenerated allocation sequence. Based on group assignment, participants will be provided with a personal access code to be entered on the PROTECT Norge study website. Entry of the code triggers the predefined scheduling of study activities within the platform.

Members of the research team involved in data analysis will be blinded to group allocation until the inferential analyses of the primary 3-month outcomes have been finalized. Study staff with direct participant contact through the PROTECT Norge help desk will not take part in data analysis.

### Intervention Fidelity

A core component of the intervention is the completion of a daily sleep diary, which supports both treatment delivery and progress monitoring. To assess adherence, participants will complete a brief questionnaire on diary compliance through their PROTECT Norge study dashboard. The PROTECT Norge infrastructure for real-time tracking of all study activities, including study activity status. Study help desk staff will manually monitor study activity in real time, including diary entries and assessment completion. These data will allow real-time monitoring of adherence. They will be asked to complete and submit this compliance log within a reasonable timeframe following the end of the intervention. For each day of the study, participants will indicate whether the diary was completed in the morning, evening, or both, with additional options for “I do not know” or “Did not complete.” This structured measure of adherence provides insight into participant engagement and supports evaluation of intervention fidelity.

### Outcome Assessment Timing

Baseline data will be obtained from all potential participants as part of the screening. At 3 months postrandomization, a similar data collection will be performed. These data will serve as the primary short-term outcome data for the study. For the waitlist group, they will also serve as an updated baseline for their intervention period, which starts immediately after they provided these data (see Textbox 1).

Textbox 1. **Overview of data collection time points.**

**Intervention arm**

- T0: Baseline
- T0 to T1: Intervention
- T1 (T0+3 mo): Follow-up (FU) 1
- T2 (T0+6 mo): FU2
- T3 (T0+12 mo): FU3

**Control arm**

- T0: Baseline
- T0 to T1: Waiting list
- T1 (T0+3 mo): FU1
- T2 (T0+6 mo): Not applicable
- T3 (T0+12 mo): Not applicable
- T0’=T1: Updated baseline
- T0’ to T1’: Intervention
- T1’ (T0’+3 mo): FU1’
- T2’ (T0’+6 mo): FU2’
- T3’ (T0’+12 mo): FU3’

### Outcomes

Study outcomes will be evaluated using validated instruments to capture the effects of the intervention on sleep, cognition, and related mental health domains through PROTECT Norge built-in clinical trials infrastructure. The primary outcome is insomnia severity, assessed using the ISI. All other outcomes are considered secondary outcomes (see Table 2 for details). Use of sleep medication is a secondary outcome and will be captured on a daily interval through the digital sleep diary.

**Table 2.** Screening instruments and outcome measures used in the study, assessed through the Platform for Research Online to Investigate Cognition and Genetics in Ageing (PROTECT) Norge infrastructure.

Short name	Long name	Details	Time frame	Assessment points
ISI	Insomnia Severity Index [25]	7 items; scale 0-28, higher scores indicate more severe insomnia symptoms.	Past 2 wk	Screening/baseline + all follow-ups
SPD	Sleep problem duration item	One item assessing duration of sleep problem, scored 0=no sleep problem to 3=3 months or more	Current	Screening
GSAQ	Global Sleep Assessment Questionnaire [32]	Four separate items scored 0-4 will be used to screen for sleep disorders	Past 4 wk	Screening
FLAME	Factors of Longitudinal Attention, Memory and Executive Function [31]	Computerized neuropsychological battery with 8 tests. Gives composite score combining subscores on domains such as attention (speed and accuracy), memory, and executive function; higher scores reflect better overall cognitive performance.	Current	Baseline + all follow-ups
PHQ-9	Patient Health Questionnaire [33]	9 items; scale 0-27, higher scores indicate more severe depressive symptoms.	Past 2 wk	Baseline + all follow-ups
GAD-7	Generalized Anxiety Disorder scale [34]	7 items; scale 0-21, higher scores indicate more severe anxiety symptoms.	Past 2 wk	Baseline + all follow-ups

### Statistical Analysis

Descriptive statistics will be presented for baseline demographics, including age, gender, ethnicity, marital status, education, and employment status. Counts and percentages (categorical variables) and either means and SDs (symmetric continuous variables) or medians and IQR (skewed continuous variables) will be presented for the total sample and per intervention arm. Similarly, descriptive statistics will be presented for primary and secondary outcome variables, for all time points.

The primary estimand will be the average treatment effect (ATE), defined in accordance with the International Council for Harmonisation (ICH) E9(R1) guideline on estimands and sensitivity analysis as the adjusted mean difference in insomnia symptoms measured as ISI scores between the intervention and waitlist arms at three months postbaseline. Nonadherence will be handled using the treatment policy strategy (which reflects the intention-to-treat principle under the estimand framework), meaning that the analysis will include all participants according to their randomized allocation, regardless of adherence.

The primary inferential analysis will thus be the comparison of the intervention and the waitlist arms at 3 months post baseline with regard to mean ISI scores. This comparison will be performed using analysis of covariance (ANCOVA), implemented as a linear regression model with follow-up ISI as the dependent variable, intervention arm as the independent variable, and baseline ISI as a covariate. The interaction between intervention arm and baseline ISI will be included to allow for slope heterogeneity. To improve precision, further adjustments will be made for potentially predictive baseline variables, ie, the demographic variables listed above plus baseline measures of depression (Patient Health Questionnaire-9) and anxiety (Generalized Anxiety Disorder-7). All continuous covariates will be entered linearly. The primary contrast will be reported as a marginal (population average) mean difference, obtained via model-based standardization over the joint baseline covariate distribution of the randomized sample.

The estimated ATE will be reported with a 95% CI based on a heteroscedasticity-robust SE and corresponding 2-sided *P* value. An effect size will be calculated by dividing the ATE by the pooled SD of baseline ISI scores.

Missing 3-month ISI and any missing baseline covariates will be handled using multiple imputation by chained equations (MICE). Imputation models will include all variables specified for the substantive model plus auxiliary variables given by 3-month measures of Patient Health Questionnaire-9, Generalized Anxiety Disorder-7, and FLAME. A total of *m*=100 imputed datasets will be generated, using logistic regression for dichotomous variables and otherwise predictive mean matching (*k*=5). Each imputed dataset will be analyzed with the primary ANCOVA, and effect estimates and standard errors will be combined using Rubin rules.

Supplementary analyses will assess the complier average causal effect. Sensitivity to missing not at random dropouts will be explored in a tipping-point (delta) analysis, by applying additive shifts to the imputed three months ISI scores separately by arm. In case of substantial skewness of the outcome, explorative sensitivity analyses will apply either Box-Cox transformation of the outcome or other regression models.

For the assessment of long-term effects on insomnia symptoms, there is no control group; thus, no conclusions will be drawn regarding treatment effects. The longitudinal development of ISI scores from baseline to 3, 6, and 12 months will be studied in linear mixed models, including random intercepts and slopes to allow for correlation between repeated measurements. Data from the original intervention arm will be combined with the (3 mo + delayed) data from the original waitlist arm for these analyses. Time since baseline (updated baseline for the waitlist arm) will be treated as a categorical variable.

Secondary outcomes will be analyzed similarly to ISI scores, with assessment of treatment impact at three months and longitudinal descriptive analysis of long-term trajectories, including covariance with insomnia symptoms. However, all secondary analyses will be exploratory.

### Power Analysis

At present, more than 3500 active participants with an active consent for contact in the PROTECT cohort. With the planned expansion, approximately 5000 participants are expected to be available at study initiation. Assuming that 15% of these (approximately 750 individuals) will meet eligibility criteria, we aim to recruit 500 participants (approximately 67% of those eligible). Allowing for 20% attrition in both arms prior to the 3-month follow-up, we expect 400 participants to be available for the primary efficacy analysis (200 per arm). This sample size provides more than 80% power to detect a small-to-medium effect size of 0.3 using a 2-sided significance level of α=.05. Applying ANCOVA in the primary analysis is expected to further increase statistical power, depending on the proportion of variance explained (*R*^2^) by baseline variables.

### Ethical Considerations

This project will be conducted in accordance with the Declaration of Helsinki [35] and complies with the General Data Protection Regulation (GDPR, 2018) and Stavanger University Hospital’s data security policy.

The ASLEEP RCT will be conducted as a nested study within the PROTECT Norge infrastructure, which was launched in 2020 and approved by the Regional Committee for Medical and Health Research Ethics, West (REK vest, reference 2019/478). A separate application for ethical approval for the ASLEEP project will be submitted to REK, and internal approval will be sought at Stavanger University Hospital, including a data protection impact assessment for any new technology used to process participant data. Only participants who have previously provided broad consent (C4C) for future studies within PROTECT Norge will be invited, and all will provide additional written informed consent specific to the ASLEEP RCT study through a secure and validated procedure. Participation is voluntary, and participants may withdraw from the study at any time, without giving a reason. No financial compensation will be provided for participation.

All study data will be stored on secure cloud servers in Norway, with regular backups and monthly data transfers from the PROTECT Norge infrastructure to internal servers at Stavanger University Hospital. Any significant protocol modifications (eg, changes to eligibility criteria, outcomes, or analyses) will be submitted for review by the REK, updated in the trial registry and appropriate internal registries at Stavanger University Hospital. Personally identifiable information will be stored separately from research data, and data will be retained for 10 years following study completion and then permanently anonymized by deleting the reidentification key. Access to personally identifiable information will be restricted to authorized research personnel only.

Specifically for the phase 2 RCT, access to identifiable data will be restricted to authorized study staff with direct participant contact through the PROTECT Norge help desk via a secure, access-controlled management portal requiring authenticated login. Pseudonymized datasets will be used for data analysis by members of the research team involved in data analysis. Study staff responsible for system administration, participant support, and adherence monitoring may have access to operational study data but will not take part in data analysis.

Data quality will be ensured through standard platform-based validation procedures and institutional quality assurance routines.

A waitlist-controlled design has been chosen to ensure that all participants will ultimately receive access to the intervention while enabling robust evaluation. Given the low-risk nature of this behavioral, fully digital intervention, no physical harms are anticipated, but depression symptoms will be regularly monitored to identify any signs of distress or harmful behavior, with follow-up procedures in place to provide appropriate support if needed.

### Patient and Public Involvement

Patient and public involvement (PPI) is a guiding pillar at the SESAM and shaping both the design and execution of research. To strengthen this commitment, SESAM established WiseAge in 2015, a dedicated platform that promotes meaningful PPI across all projects, from initial idea to full implementation. The overarching goal is to enhance the quality and relevance of research by grounding it in real-world need [36,37]. To support this, SESAM and WiseAge have developed a practical guideline for embedding PPI throughout the research process, aimed at professionals, researchers, and user representatives alike [38].

In accordance with these guidelines, the ASLEEP project has been presented to the WiseAge community panel, a forum of 15 dedicated members, ensuring broader user dialogue and input. In addition, two user representatives have been part of the ASLEEP project group from the very beginning. Their contributions have been substantial, ranging from feedback on study documents, funding applications, video materials, and sleep reports, to active participation in the development of the ASLEEP basic course and UAT reported in a previous study (JA Aakre, PhD; I Testad, PhD; MT Gjestsen, PhD; et al; unpublished data; November 2025) and dissemination. Building on this, users will remain actively engaged in Phase 1 of the ASLEEP project by being engaged in the workgroup responsible for refining the intervention and designing the delivery software in line with the human-centered design approach. In Phase 2, they will continue their established role by contributing to UAT of the nested RCT conducted on the PROTECT Norge infrastructure, while also supporting project dissemination and recruitment, ensuring continuity of user-centered input throughout the project.

## Results

The ASLEEP project started in January 2026, with funding awarded. Under the funding scheme, the DAM Foundation requires the project to be funded and published as a registered report. This paper represents the stage 1 registered report, and the results will be reported in a stage 2 registered report. As of February 2026, intervention development has commenced, initiated by a multistakeholder prototyping process involving user representatives, members of the research group, and representatives from the hospital’s Information and Communication Technology (ICT) and Innovation Departments. Subsequently, integration of the ASLEEP study as a nested digital trial within PROTECT Norge will follow. This entails establishing fully digital registration, consent, and screening procedures, updating web page content and study documentation, and scheduling questionnaires for baseline and follow-up assessments for both trial arms. Phase 1 is scheduled for completion in Q4 2027, delivering a fully developed intervention and trial infrastructure in preparation for Phase 2.

Phase 2 will commence in Q4 2027 with implementation of the ASLEEP intervention, recruitment for the RCT, and trial delivery, which will be concluded in 2028 before short-term (3-mo) analysis and subsequent dissemination through stage 2 registered reports. PPI activities have informed the ASLEEP project from its inception, and outcomes of this involvement will be reported.

A dissemination plan will be developed to share the results through relevant channels.

## Discussion

### Expected Impact

The ASLEEP project represents an innovative, tiered intervention to promote sleep health in midlife and older adults, evaluated as a fully digital, nested RCT within the PROTECT Norge research infrastructure. Sleep health is a multidimensional construct encompassing adequate quality, timing, duration, and regularity of sleep, essential for overall health and well-being [39]. By targeting insomnia symptoms in older adults, ASLEEP aims to address a highly prevalent problem in midlife and older adulthood [3] with significant negative implications for quality of life, physical and mental health [4]. The tiered design has the potential to empower individuals with the capacity and interest to take an active role in prevention, treatment, and follow-up, supporting greater self-management of their sleep health.

Grounded in CBT-I, the project will build further on an existing basic course, which delivers foundational psychoeducation, and will develop an advanced course for individuals experiencing persistent insomnia symptoms. This advanced course will be designed for delivery via a dedicated app or web platform, ensuring accessibility across devices and integration into participants’ daily routines.

The ASLEEP intervention will be evaluated in a nested clinical trial conducted entirely online through the PROTECT Norge platform, using a 2-arm, waitlist-controlled RCT design. The PROTECT Norge help desk will provide technical support, reminders, and study-related communication to minimize barriers to participation and enhance engagement. Embedding the trial within PROTECT enables efficient recruitment, centralized data collection, and long-term follow-up while supporting broad participation from across Norway, including rural and remote areas.

The RCT will evaluate both short- and long-term effects on insomnia symptoms and examine potential benefits for medication use, mental health, cognition, and brain health.

### Patient and Public Involvement

PPI has been integral to the ASLEEP project so far, with WiseAge panel input and user representatives embedded in the team to strengthen relevance and usability. These measures have shaped existing study materials, intervention design, and platform development and will continue in line with SESAM’s PPI guidelines going forward. What worked well and what did not will be reported, so others can learn from this experience.

### Limitations

Although the digital format of the ASLEEP study enables wide reach, adherence to treatment recommendations may be a challenge, as fully automated digital CBT-I programs have shown substantial variation in completion and adherence rates [40]. To address potential challenges with adherence, retention, and data completeness, a 20% dropout rate has been accounted for, and the PROTECT Norge help desk will provide proactive and continuous technical and procedural support, reminders, and guidance to encourage participant engagement throughout the trial.

Recruitment of 400 participants for randomization is ambitious given the current PROTECT Norge research willing cohort of approximately 3500 participants. While this represents a substantial recruitment pool, achieving the target sample size may be challenging; however, the ongoing expansion of the cohort toward 9000 registered participants, in line with PROTECT Norge’s established recruitment plan, is expected to strengthen recruitment feasibility.

Participation in PROTECT Norge requires a baseline level of digital competence, which supports delivery of this fully digital intervention but may limit generalizability to the broader population aged 50+ years, particularly those with less confidence in technology or internet use. However, a central element of the ongoing PROTECT Norge cohort expansion is a deliberate effort to broaden cohort diversity through targeted outreach to underrepresented demographic groups, recruitment strategies that extend beyond social media, and partnerships with community organizations and primary care providers. In addition, the PROTECT Norge help desk offers technical support intended to facilitate participation among individuals with lower digital competence. These efforts aim to mitigate selection bias in future phases of the research.

## Supplementary material

10.2196/81542 Checklist 1 GRIPP 2-SF checklist.

10.2196/81542 Checklist 2 SPIRIT 2025 checklist.

## Abbreviations

ANCOVA: analysis of covariance
ASLEEP: Preventing and Treating Insomnia Symptoms in Midlife and Older Adults
ATE: average treatment effect
C4C: consent for contact
CBT-I: cognitive behavioral therapy for insomnia
GDPR: General Data Protection Regulation
GRIPP 2-SF: Guidance for Reporting Involvement of Patients and the Public–Short Form
ICH: International Council for Harmonisation
ISI: Insomnia Severity Index
MRC: Medical Research Council
PPI: patient and public involvement
PROTECT Norge: Platform for Research Online to Investigate Cognition and Genetics in Ageing Norge
RCT: randomized controlled trial
REK: Regional Committee for Medical and Health Research Ethics
SESAM: Centre for Age-Related Medicine
SPIRIT: Standard Protocol Items: Recommendations for Interventional Trials
UAT: user acceptance testing
