# Supplementary material for: Preventing and Treating Insomnia Symptoms in Midlife and Older Adults (ASLEEP): Protocol for a Randomized Controlled Trial Using the PROTECT Norge Infrastructure
Source: JMIR Res Protoc. 2026 Mar 2;15:e81542. doi: 10.2196/81542 (PMC12954720; doi:10.2196/81542)
Supplement: Checklist 1 [file resprot-v15-e81542-s001.pdf]

From: GRIPP2 reporting checklists: tools to improve reporting of patient and public involvement in research

Section and topic	Item	Reported on page No
1. Aims	Report the aim of PPI in the study	12
2. Methods	Provide a clear description of methods used for PPI in the study	12-13
3. Study Results	Outcomes: Report the results of PPI in the study, including both positive and negative outcomes	13
4. Discussion and conclusions	Outcomes: Comment on the extent to which PPI influenced the study overall. Describe positive and negative effects	14
5. Critical perspective	Comment critically on the PPI in the study, reflecting on the things that went well and those that did not, so others can learn from this experience	14
